# Therapeutic effects of adipose-derived mesenchymal stem cells combined with glymphatic system activation in prion disease

**DOI:** 10.1186/s13024-025-00835-y

**Published:** 2025-04-17

**Authors:** Mohammed Zayed, Yong-Chan Kim, Byung-Hoon Jeong

**Affiliations:** 1 Korea Zoonosis Research Institute, Jeonbuk National University, 820-120, Hana-ro, Iksan, 54531 Republic of Korea; 2 Department of Bioactive Material Sciences, Institute for Molecular Biology and Genetics, Jeonbuk National University, Jeonju, 54896 Republic of Korea; 3 Department of Surgery, College of Veterinary Medicine, South Valley University, Qena, 83523 Egypt; 4 Department of Biological Sciences, Andong National University, Andong, 36729 Republic of Korea; 5 School of Life Sciences and Biotechnology, Gyeongkuk National University, Andong, 36729 Republic of Korea

## Abstract

There is currently no effective therapy for prion diseases. The glymphatic system is an organized system of perivascular spaces that facilitates the removal of metabolic waste from the brain. This study demonstrates the therapeutic potential of a combination therapy of adipose-derived mesenchymal stem cells (AdMSCs) and a glymphatic system-activated drug, clonidine, against prion disease. The therapy has the potential to clear PrP^Sc^ accumulation, ameliorate astrocytosis, and prolong the survival time of ME7-infected mice.

**Supplementary Information:**

The online version contains supplementary material available at 10.1186/s13024-025-00835-y.

Prion diseases are incurable, irreversible neurodegenerative disorders caused by misfolding of the normal form of the prion protein (PrP^C^) into the pathological form (PrP^Sc^) [1]. Despite efforts to find a therapy, prion diseases pose significant challenges due to neuroinflammation and irreversible neurological damage [2, 3]. One currently available therapeutic strategy is based on compounds that can inhibit or clear PrP^Sc^ accumulation [4, 5]. Targeting the glymphatic system for the treatment of neurological diseases has emerged as a potential therapy [6]. In prion diseases, the perivascular waste-clearing system has been observed to upregulate the key protein of the glymphatic system, aquaporin-4 (AQP4) [7, 8], suggesting the therapeutic potential of the glymphatic system. We recently attempted to treat prion-infected mice using glymphatic system activation molecules, including clonidine, and found that they led to significant PrP^Sc^ clearance [8]. However, it did not show a substantial increase in survival time, probably due to the neurotoxicity induced by early PrP^Sc^ deposition. Mesenchymal stem cells (MSCs) have been used as a potential cell-based therapy for neurodegenerative disorders such as stroke, traumatic brain injury, multiple sclerosis, and Alzheimer’s and Parkinson’s diseases [9, 10]. Previous attempts were made to use MSC transplantation to treat animal models of prion infection [11–13]. Those attempts showed some recovery, including prolonged survival and reduced gliosis in prion-infected mice. However, they did not achieve a complete cure or reduce the accumulation of PrP^Sc^. A main challenge in MSC-based prion therapies is the infectivity of MSCs isolated from bone marrow or adipose tissue, as validated by tissue-derived homogenate-inoculated mice [14]. Thus, allogeneic transplantation of MSCs is highly recommended. A synergistic combination of a drug that can clear PrP^Sc^ deposition and a cell-based therapy to rescue neuronal loss and damage is required. Therefore, the therapeutic potential of the combination therapy (clonidine and adipose-derived MSCs (AdMSCs)) in ME7-infected mice was evaluated through the survival time, detection of PrP^Sc^ deposition, and amelioration of astrocytosis and microgliosis. Protein markers for neuronal loss and differentiation were also assessed.

Adipose tissue from the inguinal region of C57BL/6J mice at 6–8 weeks of age was collected for isolation of MSCs as previously shown [15, 16]. AdMSCs were characterized by their ability to adhere to the tissue culture dish, proliferation rate and forming a colony unit, trilineage differentiation, and the expression of different MSC-specific surface markers using flow cytometry analysis (Fig. S1A). To investigate the therapeutic effect of AdMSC transplantation combined with clonidine on prion disease pathologies, mice were intraperitoneally injected with the ME7 scrapie strain. All mice were inoculated on the same day. Our previous study demonstrated typical characteristics of prion disease through a reduction in body weight, abnormal behaviors, and death of ME7-infected mice [14]. Beginning 7 days post-injection (dpi), the mice received weekly clonidine treatments (100 µg/kg) as previously explained [8], which continued for 18 weeks. Cultured AdMSCs were collected and resuspended in PBS at a concentration of 1 × 10^5^ cells per 30 µl and transplanted intracranially at 70 dpi. Prior to intracranial injection, all mice were anesthetized with 2% isoflurane (Hana Pharm Co., Ltd; Seoul, South Korea) in an induction chamber. The mice were subsequently observed. The negative control (CTL) mice were injected with 30 µl of PBS. After 150 dpi, a cohort of mice (*n* = 4/group) were sacrificed for the detection of PrP^Sc^ and astrocytosis by western blotting. To perform survival analysis, the remaining mice (*n* = 6/group) were monitored and sacrificed at 373 dpi. Mice were euthanized at 373 dpi due to the development of atopic dermatitis. Mice in the CTL group, which also developed atopic dermatitis, were sacrificed after all mice in the ME7 and treated groups had been euthanized. Figure 1A provides a schematic diagram of the study design. ME7-infected mice treated with the combination therapy of clonidine and AdMSCs did not show any disease symptoms for up to 373 dpi, unlike non-treated ME7-infected mice (230.7 ± 1.4 dpi; *n* = 6, Fig. 1B). The misfolding and aggregation of PrP^C^ into amyloid fibrils are strongly linked with prion disease [17]. Thus, brain tissues were homogenized and examined using western blotting to identify the total PrP and PrP^Sc^ levels to establish the present treatment’s therapeutic effect. Equivalent quantities of protein (30 µg) were loaded, and the band intensity was analyzed in ImageJ. Results showed that the band intensity of total PrP from the brain samples did not differ among the three groups (Fig. 1C). Notably, the group that received combination therapy of clonidine and AdMSC transplantation demonstrated lower PrP^Sc^ expression than the non-treated ME7-infected group at 150 dpi (Fig. 1C), and the CTL mice showed no PrP^Sc^ expression. Next, we examined the expression of PrP^Sc^ at 373 dpi of the disease. A clear difference in PrP^Sc^ deposition was observed between the treated and non-treated groups (Fig. 1C).

**Fig. 1:**
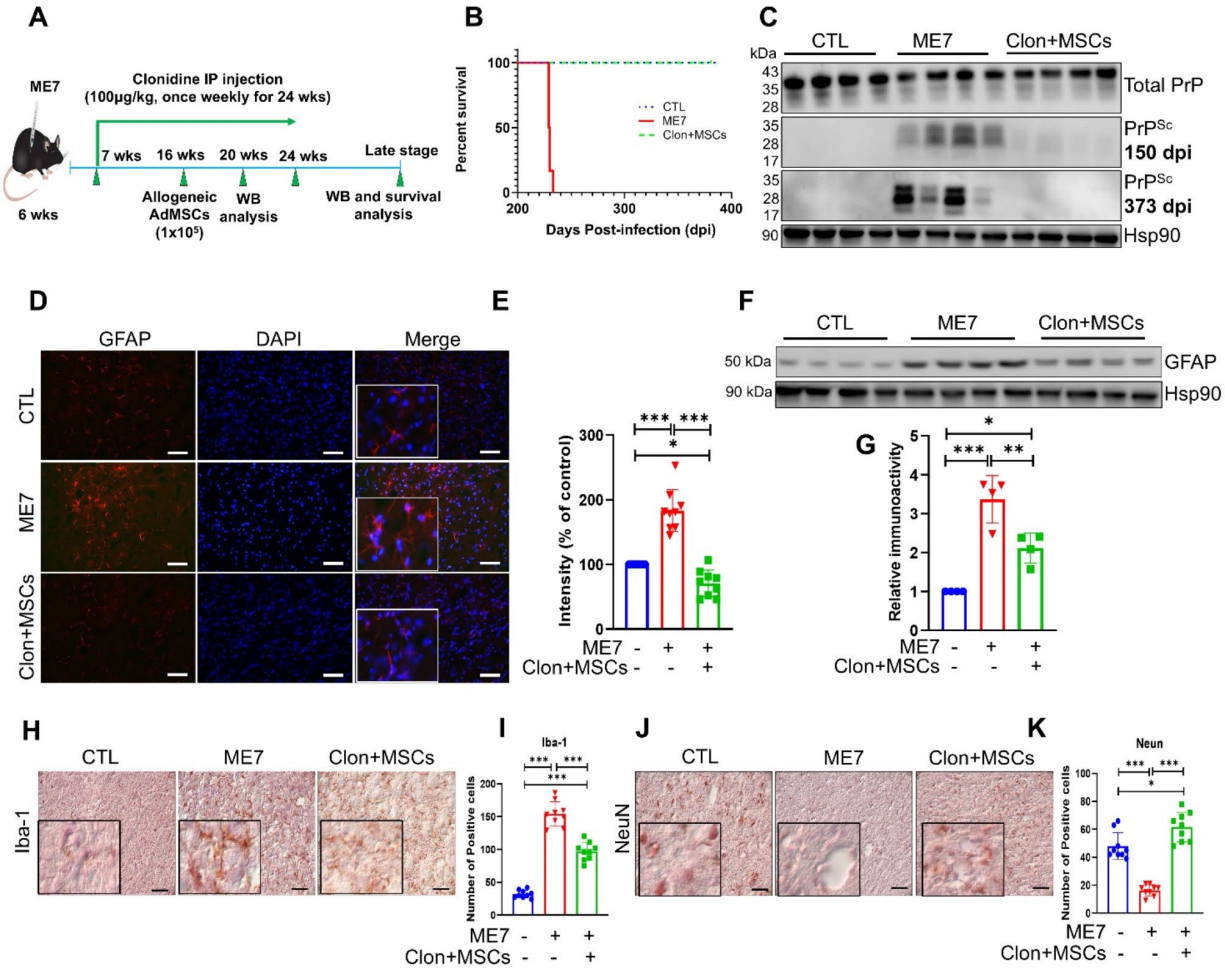
Evaluation of the therapeutic potential of combining clonidine with adipose-derived mesenchymal stem cells (AdMSCs) transplantation in ME7-infected mice. **A** Prion-infected mice were treated with clonidine (100 µg/kg) weekly and received AdMSCs once, 70 days post-infection (dpi). **B** A Kaplan- Meier survival curve was generated to show how combining clonidine and AdMSCs affected the survival of ME7-infected mice. ME7-infected mice that received the combination of clonidine and AdMSCs did not show any symptoms of the disease for up to 373 dpi, unlike the non-treated ME7-infected mice (230.7 ± 1.4 dpi; *n* = 6 per group). Thus, the combination therapy extended the average survival by at least 140 days (*p* < 0.0001, log-rank test). **C** Effects of the combination therapy on the expression of total PrP and PrP^Sc^ accumulation in ME7-infected mice at 150 and 373 dpi, as shown by western blotting. **D** Immunofluorescence micrographs of the thalamus region from mouse treated or untreated with clonidine and AdMSCs at 150 dpi. The tissues were stained with GFAP-specific antibody and developed with anti-mouse Alexa 647 (red) (*n* = 3 per group). Cell nuclei are stained with DAPI (blue). Scale bar: 50 μm. **E** Quantitative analyses of GFAP using immunofluorescence staining from the experiments panel. *p < 0.05, ***p < 0.001. **F** GFAP expression by western blotting (*n* = 4 per group) at 150 dpi. HSP90 served as a loading control. **G** Quantitative analyses of GFAP immunoblots from the experiments panel. *p < 0.05, **p < 0.01, and ***p < 0.01. **H** Photomicrographs of immunohistochemistry staining for Iba-1 in the thalamus of mice from each group at 150 dpi, scale bar: 50 μm. **I** Quantification of the number of Iba-1-positive cells. ***p < 0.001. **J** Photomicrographs of immunohistochemical staining for NeuN in the thalamus of mice from each group at 373 dpi, scale bar: 50 μm. Immunohistochemistry was used in cryosections from the three treatment groups. **K** Quantification of the number of NeuN-positive neurons in the thalamus of mice from each group (*n* = 3 per group). *p < 0.05 and ***p < 0.001. All data are shown as the mean ± SD and were analyzed using one-way ANOVA with post-hoc Tukey’s test

Inflammation involves reactive astrogliosis and microgliosis plays a significant role in the development of neuroinflammatory and neurodegenerative conditions in prion disease [18]. An increase in GFAP, an astrocyte activation marker, was linked to PrP^Sc^ in the brain tissues of prion-infected mice [18]. Therefore, we assessed the effect of the combination therapy on the level of GFAP. We examined GFAP expression by immunofluorescence of brain sections from the thalamus region and by immunoblot analysis using whole brain homogenates. The ME7 strain induces significant thalamic pathology, including neuroinflammation and neuronal degeneration [19], highlighting importance of the thalamus in prion disease. The immunofluorescence analysis showed that AdMSC transplantation with clonidine significantly decreased GFAP levels at 150 dpi, compared with the non-treated group (Fig. 1D, E; *p* < 0.001). These findings were supported by western blot analysis, which demonstrated that the protein level of GFAP in brain homogenates was significantly inhibited by the combination therapy compared with in the non-treated group (Fig. 1F, G; *p* < 0.01). Next, we tested the effect of the combination therapy on the reduction of microglial activation of ME7-infected mice brain homogenates using Iba-1, a marker for activated microglia. We found that our treatment significantly reduced the elevated level of Iba-1 in ME7-infected mice as compared to non-treated mice at 150 dpi (Fig. 1H, I; *p* < 0.001).

Finally, to examine the protective effect of the combination therapy on neurons, we evaluated the extent of neurogenesis. Our immunohistochemistry findings for beta III-tubulin and NeuN, markers of neuronal health, show that the level of NeuN (Fig. 1J, K, Fig. S2) but not beta III-tubulin (Fig. S3) increased significantly in the treated group, compared with the non-treated ME7-infected mice at 230 dpi (*p* < 0.001). Therefore, our results validate the potential of clonidine and AdMSCs as a treatment for prion and this combination is also a potentially promising therapy for other degenerative brain diseases. While our therapy proved effective in the ME7 scrapie strain, the distinct biochemical and neuropathological profiles of different prion strains may require tailored interventions [20]. Further studies using other prion strain models are needed to elucidate the specific mechanisms underlying the combination of AdMSCs with clonidine.

## Electronic supplementary material

Below is the link to the electronic supplementary material.

Supplementary Material 1

## Data Availability

The data used to generate the results that support the findings are all included in the manuscript. Source data can be requested from the corresponding author upon reasonable request.
