# Supplementary material for: Therapeutic effects of adipose-derived mesenchymal stem cells combined with glymphatic system activation in prion disease
Source: Mol Neurodegener. 2025 Apr 17;20:42. doi: 10.1186/s13024-025-00835-y (PMC12007347; doi:10.1186/s13024-025-00835-y)
Supplement: Supplementary file 1 — Supplementary Material 1 [file 13024_2025_835_MOESM1_ESM.docx]

**Supplementary information**

**Materials and methods**

**Animals**

C57BL/6J mice at 6–8 weeks of age (Nara Biotech, Pyeongtaek, Gyeonggi, Korea) were used for this study. The mice had unrestricted access to food and water. All animal experiments were approved by the Animal Care Committee at Jeonbuk National University (JBNU 2020-080) according to the guidelines issued by the international ARRIVE instructions.

**Isolation and culture of mouse AdMSCs**

Adipose tissue from the inguinal region was collected and washed with PBS (Biosesang, Yongin-si, Gyeonggi-do, Korea) for MSCs isolation as shown previously [[1](#_ENREF_1)]. In brief, the tissues were minced and digested with 0.1% type I collagenase (FUJIFILM Wako Pure Chemical Corp., Tokyo, Japan) for 30 minutes at 37°C. A basic growth medium of Dulbecco's Modified Eagle Medium (DMEM) (Gibco Life Technologies, NY, USA) supplemented with 10% fetal bovine serum (FBS) and 1% penicillin-streptomycin (Gibco) was added to stop the digestion. The solution was centrifuged at 700 × g, and the supernatant was discarded. The cell pellets were resuspended in the culture medium and cultured at 37°C in a humidified 5% CO_2_ incubator. The medium was changed every 2 days, and AdMSCs of passages P2–P4 were used. Cells were detached and passaged at 80% confluence using the TrypLE Select enzyme (Gibco). For the colony forming unit (CFU) assay, AdMSCs derived from 3 different mice were seeded onto 100-mm cell culture dishes at a density of 1,000 cells per dish. The cells were cultured for 10 days, and colonies were fixed and stained with crystal violet for 30 min. The proliferation rate of the AdMSCs was measured using a Cell Counting Kit-8 (CCK-8, Cambridge, UK) according to the manufacturer’s instructions. Briefly, AdMSCs (n = 3) were seeded onto a 96-well plate (5000 cells/well) and cultured for 24, 48, and 72 h before the addition of 10 μl of cell counting solution. The absorbance at 450 nm was measured using a microplate spectrometer (Molecular Devices, California, USA).

**Multilineage profile of AdMSCs**

AdMSCs were induced to differentiate into adipocytes, chondrocytes, and osteocytes as previously described [[2](#_ENREF_2)] with slight modifications. Adipogenic differentiation was induced in basic growth medium supplemented with 1 µmol/l dexamethasone (Sigma-Aldrich, Gangnam-gu, Seoul, Korea), 10 µg/ml recombinant human insulin (Sigma-Aldrich), 10 µmol/l L-ascorbic acid (Sigma-Aldrich), and 20 µmol/l indomethacin (Sigma-Aldrich) for 10 days. The chondrogenic differentiation medium was supplemented with 100 nmol/l dexamethasone, 0.5 mmol/l L-ascorbic acid (Sigma-Aldrich), and 10 ng/ml transforming growth factor beta 1 (TGF β1, R&D Systems, Minneapolis, MI, USA) for 14 days. The osteogenic differentiation medium was supplemented with 100 nmol/l dexamethasone, 10 mmol/l β-glycerophosphate (Sigma-Aldrich), and 0.1 mmol/l L-ascorbic acid (Sigma-Aldrich) for 21 days. The presence of adipocytes was determined using 0.5% Oil Red O staining (Fisher Scientific, Pittsburgh, PA, USA), osteoblasts by 2% alizarin red staining (Fisher Scientific), and chondrocytes by alcian blue (pH = 2.5) (Fisher Scientific). The stained cells were visualized and photographed using a phase contrast microscope (Carl Zeiss, Oberkochen, Germany).

**Flow cytometry**

AdMSCs were subjected to a flow cytometric analysis using a cluster-of-differentiation (CD) antigen–antibody panel of FITC‐labeled anti-mouse CD105, CD90, CD44, and CD34 (BD Bioscience, Franklin Lakes, NJ, USA), as previously reported [[3](#_ENREF_3)] (for details, see supplementary file).

**Experimental prion-infected mice and treatment**

The prion disease model was constructed as previously described [[4](#_ENREF_4)] using an intraperitoneal injection of 100 μl of 1% (w/v) brain homogenate prepared from terminally ill ME7 scrapie-infected mice. To prepare AdMSCs, cultured AdMSCs at P2 were washed 3 times with PBS, collected with TrypLE Select enzyme, and resuspended in PBS at a concentration of 1×10^5^ cells per 30 μl. Clonidine (100 μg/kg, Sigma-Aldrich) was dissolved in 1X PBS as previously explained [[4](#_ENREF_4)].

Beginning 7 days post-infection (dpi), clonidine (100 μg/kg) was administered weekly for 18 weeks, and AdMSCs (1×10^5^) were administered intracranially at 70 dpi. Intracranial injection was done by free-hand injection technique with a syringe, guided by anatomical landmarks like the lambda suture and eyes. For the negative control, mice were injected with 30 μl of PBS. Mice inoculated with only the ME7 scrapie strain were kept as a positive control. Our previous study demonstrated typical characteristics of prion disease in ME7-infected mice, including progressive weight loss, abnormal behaviors, and eventual mortality [[5](#_ENREF_5)]. After 7 months, the mice were sacrificed for the detection of PrP^Sc^ and astrocytosis by western blotting (n = 4/group). To perform survival analysis, the mice were observed and sacrificed at a late stage (n = 6/group). Mice in the negative control group were sacrificed after all ME7-infected and treated mice had been euthanized.

**Preparation of brain tissue and western blot analysis**

The brain was collected and homogenates were prepared according to our previously published protocol [[4](#_ENREF_4)]. Brains were collected to evaluate proteinase K-resistant PrP^Sc^. To detect PrP^Sc^ in the brain, 40 µg/ml proteinase K was added to quantified samples for 1 h at 37°C. The proteinase K–treated samples were then heated to 95°C for 10 min in 5x sample buffer (Thermo Fisher Scientific, Waltham, USA). For the western blot analysis, 20% brain homogenates were analyzed with a BCA Protein Assay Kit (Thermo Scientific) to quantify protein concentration. A total of 30 μg of protein from different groups was loaded and separated on a 12% polyacrylamide gel. The gel was electroblotted onto a PVDF membrane (Amersham, Little Chalfont, UK) at 100 V for 90 min. The membrane was then blocked in 5% skim milk for 2 h. For each analysis, the following antibodies were incubated overnight at 4°C to identify the intended protein: SAF84 (1:200, Cat#: A03208, Bertin, Montigny le Bretonneux-France), glial fibrillary acidic protein (GFAP) (1:200, Cat#: sc-33673, Santa Cruz Biotechnology, Dallas, Texas, USA), and purified mouse anti-Hsp90 (1:100, Cat#: 610419, BD Transduction Laboratory). Antigens were detected using anti-mouse secondary antibodies (Sigma-Aldrich). Immunoreactive bands were visualized with a Pierce ECL kit (Thermo Fisher Scientific, Waltham, USA). Protein levels were normalized to Hsp90 for quantification. ImageJ software (NIH, Bethesda, MD, USA) was used for the densitometric analysis of immunoblots.

**Immunohistochemistry and immunofluorescence analyses**

For immunohistochemical staining, brain tissue was fixed in 4% paraformaldehyde solution for 24 h, incubated in 30% sucrose solution, and then processed in cryo-embedding medium and optical cutting compound. The embedded tissues were sliced to 20 µm using a cryomicrotome (Thermo Fisher Scientific, Waltham, USA), mounted on slides, and stored at -20°C. Sections were treated with 0.5% Triton X-100 (Sigma-Aldrich) for 15 min and blocked with antibody blocker/diluent (Enzo Biochem, Inc. New York, USA) for 1 h at room temperature (RT). The slides were treated with primary antibody overnight in a humidity chamber at 4°C. The following primary antibodies were used: anti-beta III tubulin (Cat#: ab52623), NeuN (Cat#: ab177487), and Iba-1 (Cat#: ab283319) (all from Abcam, Cambridge, UK). Tissue was washed and incubated with secondary antibodies for 1 h followed by development with 1X HighDef^TM^ IHC chromogen substrate and working solutions (Enzo Biochem, Inc.) until a color change was observed. Hematoxylin was used as the counterstain. Representative images were taken using a Zeiss Axio-Imager M2 microscope equipped with an Axiocam 506 color camera. Positive cells in the sections were quantitatively analyzed using ImageJ software (National Institute of Health, Bethesda, MD, USA <https://imagej.net/ij/download.html>. To minimize bias, at least three randomly selected microscopic fields per section (n = 3) were analyzed.

For immunofluorescence analysis, brain sections from the thalamus region were prepared as described above. GFAP (Santa Cruz Biotechnology) at 1:50 dilution was incubated overnight in a humidity chamber at 4°C after being blocked with a power block (BioGenex, CA, USA). After being washed with phosphate-buffered saline with Tween, the slides were incubated for 1 h with an anti-mouse Alexa Fluor-647 (Cell Signaling, #4414) secondary antibody. The slides were washed and coverslipped with Prolong Gold Antifade mounting medium (Invitrogen, MA, USA) and imaged on a Zeiss Axio-Imager M2 microscope. All images were acquired using the same settings. Data analyses of fluorescence intensity were performed with ImageJ software (NIH, Bethesda, MD, USA).

**Statistical analysis**

Statistical analyses used Student's t-test and one-way analysis of variance (ANOVA) with post-hoc Tukey testing and were performed in SPSS 25.0 (IBM, Armonk, NY). The figures show statistical significance at the following *p*-values: * *p*< 0.05, ** *p*< 0.01, and *** *p*< 0.001. All results from triplicate experiments are shown as the mean ± standard deviation (SD).


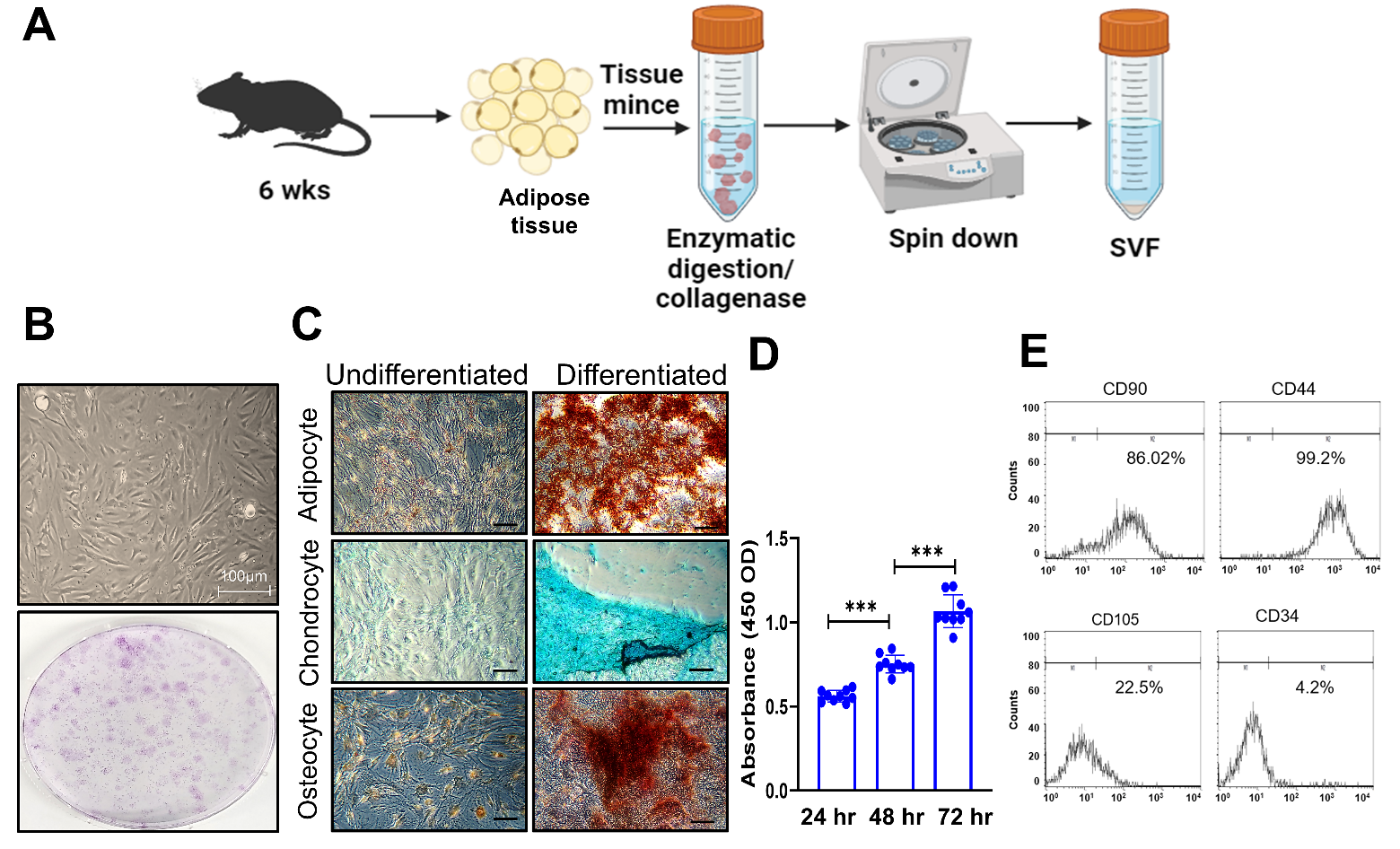


**Fig. S1** Isolation and identification of adipose-derived mesenchymal stem cells (AdMSCs). **A** Overview schematic detailing the main steps in isolating AdMSCs, stromal vascular fraction (SVF). **B** Cellular morphology (upper panel) and colony-forming units of AdMSCs stained with crystal violet (lower panel). **C** AdMSCs were positive for Oil Red O (adipogenic differentiation), alizarin red S (osteogenic differentiation), and alcian blue (chondrogenic differentiation) (scale bars: 100 μm). Corresponding representative images for undifferentiated cells are provided (scale bars: 100 μm). **D** CCK-8 assay was used to evaluate the rate of proliferation in AdMSCs over 3 days. Absorbance and optical density (OD) at 490 nm are linearly related to the number of cells and thus represent the proliferation at a given time point. *** *p* < 0.001. All data are expressed as the means ± SD (n=3). Statistical testing was done by one-way ANOVA with post-hoc Tukey’s multiple comparisons test. **E** Characterization of AdMSCs with flow cytometry through an immunophenotype analysis of MSC cell surface markers (CD90, CD44, CD105) and a hematopoietic marker (CD34) (n=3). Graphic created with BioRender.com.

**B**


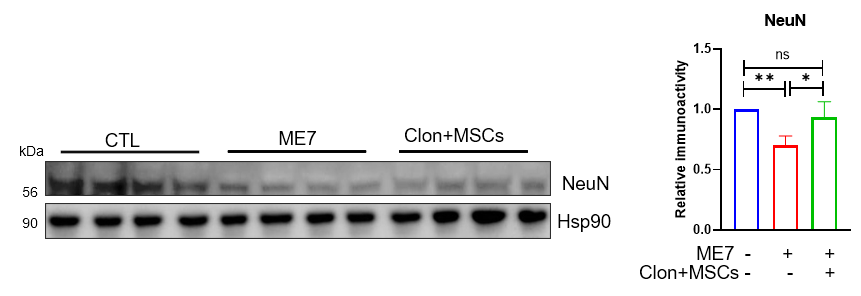


**A**

**Fig. S2 A** NeuN immunoblots by western blotting (n=4 per group) at 373 dpi. HSP90 served as a loading control. **B** Quantitative analyses of NeuN immunoblots from the experiments panel. **p* < 0.05 and ***p* < 0.01. ns= non-significant.


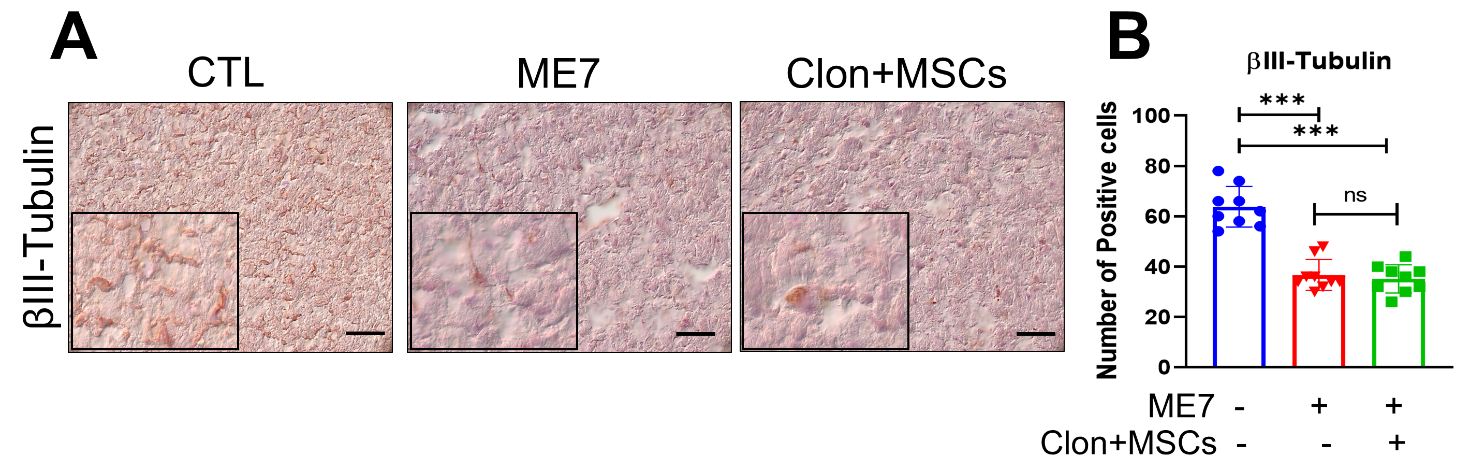


**Fig. S3** **A** Photomicrographs of immunohistochemistry staining for βIII-tubulin in the thalamus of mice from each group at 373 dpi, scale bar: 100 μm. Immunohistochemistry was used in cryosections from the three treatment groups. **B** Quantification of the number of βIII-tubulin-positive neurons in the thalamus of mice from each group (n=3 per group) (****P* < 0.001). All data are shown as the mean ± SD and were analyzed using one-way ANOVA with post-hoc Tukey’s test. ns= non significant.

**References for the supplementary file**

1. Zayed M, Jeong BH: Adipose-Derived Mesenchymal Stem Cell Secretome Attenuates Prion Protein Peptide (106-126)-Induced Oxidative Stress via Nrf2 Activation. Stem Cell Rev Rep 2024.

2. Zayed MN, Schumacher J, Misk N, Dhar MS: Effects of pro-inflammatory cytokines on chondrogenesis of equine mesenchymal stromal cells derived from bone marrow or synovial fluid. Vet J 2016, 217**:**26-32.

3. Zayed M, Caniglia C, Misk N, Dhar MS: Donor-Matched Comparison of Chondrogenic Potential of Equine Bone Marrow- and Synovial Fluid-Derived Mesenchymal Stem Cells: Implications for Cartilage Tissue Regeneration. Frontiers in Veterinary Science 2017, 3.

4. Kim Y-C, Won S-Y, Jeong B-H: Altered expression of glymphatic system-related proteins in prion diseases: Implications for the role of the glymphatic system in prion diseases. Cellular & Molecular Immunology 2021, 18**:**2281-2283.

5. Sim H-J, Kim Y-C, Bhattarai G, Won S-Y, Lee J-C, Jeong B-H, Kook S-H: Prion infection modulates hematopoietic stem/progenitor cell fate through cell-autonomous and non-autonomous mechanisms. Leukemia 2023, 37**:**877-887.
